# Molecular Mechanism of the UDP-Glucuronosyltransferase 2B20-like Gene (*AccUGT2B20-like*) in Pesticide Resistance of *Apis cerana cerana*

**DOI:** 10.3389/fgene.2020.592595

**Published:** 2020-11-19

**Authors:** Xuepei Cui, Chen Wang, Xinxin Wang, Guilin Li, Zhenguo Liu, Hongfang Wang, Xingqi Guo, Baohua Xu

**Affiliations:** ^1^College of Animal Science and Technology, Shandong Agricultural University, Tai’an, China; ^2^State Key Laboratory of Crop Biology, College of Life Sciences, Shandong Agricultural University, Tai’an, China

**Keywords:** UDP-glucuronosyltransferases, *Apis cerana cerana*, pesticide resistance, RNAi, oxidative stress

## Abstract

UDP-glucuronosyltransferases (UGTs), being multifunctional detoxification enzymes, play a major role in the process of resistance to various pesticides in insects. However, the mechanism underlying the molecular regulation of pesticide resistance remains unclear, especially in *Apis cerana cerana*. In this study, all of the UGTs in *Apis cerana cerana* (AccUGT) have been identified through the multiple alignment and phylogenetic analysis. Expression of AccUGT genes under different pesticides, and antioxidant genes after silencing of *AccUGT2B20-like*, were detected by qRT-PCR. The resistance of overexpressed *AccUGT2B20-like* to oxidative stress was investigated by an *Escherichia coli* overexpression system. Also, antioxidant-related enzyme activity was detected after silencing of the *AccUGT2B20-like* gene. Expression pattern analysis showed that almost all UGT genes were upregulated under different pesticide treatments. This result indicated that AccUGTs participate in the detoxification process of pesticides. *AccUGT2B20-like* was the major gene because it was more highly induced than the others. Overexpression of *AccUGT2B20-like* in *E. coli* could effectively improve oxidative stress resistance. Specifically, silencing the *AccUGT2B20-like* gene increased oxidative stress by repressing the expression of oxidation-related genes, decreasing antioxidant-related enzyme activity, and increasing malondialdehyde concentration. Taken together, our results indicate that AccUGTs are involved in pesticide resistance, among which, *AccUGT2B20-like* contributes to the detoxification of pesticides by eliminating oxidative stress in *Apis cerana cerana*. This study explains the molecular basis for the resistance of bees to pesticides and provides an important safeguard for maintaining ecological balance.

## Introduction

The *Apis cerana cerana* (*A. cerana cerana*) is an important pollinator that plays a critical role in maintaining the balance of regional ecologies in China. However, recently, the survival of *A. cerana cerana* has been seriously threatened because of pressure from multiple sources, including excessive use of pesticides, heavy metal pollution, and epidemics of honeybee diseases (Gegear et al., 2006; Li et al., 2012). It appears certain that exposure to these multiple stressors is the driving force behind *A. cerana cerana* colony losses (Goulson et al., 2015). Among these stressors, pesticides are the most controversial and debated. Extensive research has resulted in the detection of 161 different pesticides in honey bee colonies, and bees are often chronically exposed to pesticides throughout their development and adult life (Marie-Pierre et al., 2006; Krupke et al., 2012; Sanchez-Bayo et al., 2014). Several insecticides, such as neonicotinoids, are neurotoxins that target the central nervous system of insects, bind to postsynaptic nicotinic acetylcholine receptors, and can result in overstimulation, paralysis, and death (Tomizawa and Casida, 2004).

Considerable evidence has revealed a strong correlation between the levels of oxidative stress, reactive oxygen species (ROS) generation, antioxidase, and various types of toxicity associated with pesticides (Wang et al., 2018a). Oxidative stress occurs as a result of inadequate antioxidant defense or overproduction of free radicals and is initiated by ROS (Adams et al., 2015; Swomley and Butterfield, 2015). Once the honeybees are affected by the pesticides, the pesticides may induce oxidative stress leading to ROS or reactive nitrogen species generation and related toxic effects. Oxidative stress can increase the antioxidant defense system and lead to damage of cellular macromolecules, such as DNA, lipids, and proteins (Weidinger and Kozlov, 2015). Following oxidative stress, cell death can occur via apoptosis or necrotic mechanisms. Normally, the body maintains a steady balance between ROS generation and scavenging, which results in a relatively low level of intracellular ROS. However, stress caused by pesticides disrupts homeostasis and leads to excessive production or accumulation of ROS. To defend against oxidative damage, organisms have evolved complex mechanisms to maintain cellular homeostasis (Zhang et al., 2013). UDP-glucuronosyltransferases (UGTs) are a part of critical detoxification mechanisms (Hasegawa et al., 2010; Ahn et al., 2012). However, whether UGTs are involved in the regulation of oxidative stress caused by pesticides remains unknown.

UGTs (EC 2.4.1.17), being the major enzyme in the reaction of glycosylation, catalyze the conjugation of a diverse range of small lipophilic compounds with sugars to produce glycosides (Bock, 2003; Lairson et al., 2008; Ahn et al., 2012). UGTs represent major phase II drug-metabolizing enzymes that process and detoxify a large number of important endogenous compounds, drugs, and environmental agents (Mazerska et al., 2016). UGTs function by transforming their substrates into more polar metabolites, which are easier to remove from the cell by export transporters (Mazerska et al., 2016). The structure of a protein determines its biological function. The majority of UGT proteins are in the endoplasmic reticulum lumen (ER) and are composed of two functional domains, the N-terminal and C-terminal domains (Meech and Mackenzie, 1998). The N-terminal aglycone substrate-binding domain has a signal peptide responsible for targeting the membrane to the ER compartment. The C-terminal transmembrane domain anchors the protein to the ER membrane (Magdalou et al., 2010). The C-terminal is more conserved than the N-terminal. The UGT motif signature, (FVA)-(LIVMF)-(TS)-(HQ)-(SGAC)-G-X(2)-(STG)-X(2)-(DE)-X(6)-P-(LIVMFA)-(LIVMFA)-X(2)-P-(LMVFIQ)-X(2)-(DE)-Q, is at the C-terminal of different UGTs (Mackenzie et al., 1997; Ahn et al., 2012). UGTs, as a superfamily, have evolved in all kingdoms of life. Although the structure and functions of UGTs have been investigated in different species, UGTs still have specificity in different species.

UGTs are ubiquitous in all free-living organisms, including bacteria, fungi, plants, and animals (Bock, 2016). As major phase II enzymes in a detoxification system, UGTs catalyze the conjugation of a sugar, donated by a UDP-glycoside, typically to a lipophilic molecule, generating water-soluble products that can be easily excreted, as well as stably managed. Therefore, they can protect the cellular system from being damaged by toxic foreign compounds and regulate internal molecules more easily (Bock, 2003). In humans, the substrates for phase II metabolic enzymes are not only xenobiotics but also many endogenous compounds that play crucial functions in organisms. UGTs are involved in the conjugation of a variety of xenobiotics, including the phytoalexin quercetin and plant flavonoids, which are significant antioxidants in the human diet (Achour et al., 2014). In the process of catalyzing endogenous compounds, UGT8A1 is involved in the conjugation of ceramides and bile acids with galactose (Meech et al., 2015). UGT3A1 and UGT3A2 play crucial roles in the conjugation with N-acetylglucosamine and glucose, respectively (Meech et al., 2012). Previous studies have indicated that the glycosylation of small lipophilic compounds has long been considered an enzymatic detoxification mechanism in insects (Brattsten, 1988; Després et al., 2007). Li X. et al. (2018) provided evidence that the expression level of UGTs in *Plutella xylostella* was highly induced by more than five insecticides, whereas indoxacarb and metaflumizone significantly repressed the expression of most UGT genes. In *Aphis gossypii*, inhibitors of UGT enzymes significantly increased the toxicity of thiamethoxam against the resistant strain, and suppression of UGT transcripts by RNAi increased mortality (Pan et al., 2018). *GST-4* and *UGT-13* expression could be induced by low concentrations of allyl isothiocyanate in nematodes. This suggests that allyl isothiocyanate might confer tolerance to the nematode against oxidative stress, and their lifespan was considerably extended under the paraquat treatment (Hasegawa et al., 2010). Although many studies have been conducted on the function of UGTs in animals, there are still few published data on insects. To date, no research has been conducted on *A. cerana cerana*. Therefore, understanding the role of UGTs in resistance against pesticides could provide a basis for studying the detoxification system and a better understanding of the molecular regulation mechanism of UGTs in *A. cerana cerana* (AccUGTs).

In this study, we aim to investigate the molecular mechanism of AccUGTs under different pesticide treatments. We identified 10 AccUGT genes based on genome data. The phylogenetic relationships and tissue-specific expression profiles of these UGT genes were analyzed. Based on the expression profiling of AccUGT genes under different pesticide treatments, the *AccUGT2B20-like* gene was selected as the critical gene. Overexpression of *AccUGT2B20-like* in *E. coli* enhanced resistance to oxidative tolerance. Silencing of *AccUGT2B20-like* in adult bees reduced their ability to regulate oxidative stress. These results broadened our knowledge regarding the biological roles of UGTs in pesticide resistance and significantly improved our understanding of the relationship between UGTs and oxidation resistance in *A. cerana cerana*.

## Materials and Methods

### Insects and Treatments

*A. cerana cerana* used in this study were obtained from the experimental base of Shandong Agricultural University (Tai’an, China). Fifteen-day-old worker bees were collected from the outdoor hives by marking newborn worker bees with paint 15 days before collection. Experimental honeybees were randomly placed into wooden cages, and maintenance was performed as previously described (Wang et al., 2014). The bees were divided into eight groups (*n* = 50/group). Each group was injected with 1 μl of different pesticides between the second and third abdominal segments of the bees. The pesticides included paraquat (0.6 mg/L), DDV (0.775 mg/L), acetamiprid (0.016 mg/L), lambda-cyhalothrin (0.012 mg/L), emamectin-benzoate (0.003 mg/L), mancozeb (0.002 mg/L), and abamectin (0.003 mg/L). The oxidative stress treatment consisted of H_2_O_2_ (0.5 μl/ml). Honeybees were collected at 0, 0.5, 1, 1.5, 2, or 2.5 h after treatment. Honeybees treated at 0 h were used as controls. The treated samples were immediately snap-frozen in liquid nitrogen and stored at −80°C.

For tissue-specific expression profile analysis, different kinds of tissues (head, antenna, muscle, feet, midgut, rectum, and cuticula) were dissected from 15-day-old worker bees on ice, frozen in liquid nitrogen, and stored at −80°C for total RNA isolation.

### RNA Extraction and Quantitative Real-Time PCR

Total RNA for first-strand complementary DNA (cDNA) synthesis was extracted from experimental honeybees using the method described by Wang (Wang et al., 2018b). RNA was reverse transcribed into cDNA using 5 × All-In-One RT MasterMix with the AccuRT Genomic DNA Removal Kit (Applied Biological Materials Inc., Richmond, British Columbia, Canada), which facilitated the elimination of contaminating genomic DNA from RNA. qRT-PCR was performed using SYBR Premix Ex Taq (Takara) in 20-μl reaction volumes on a CFX96TM Real-time Detection System (Bio-Rad). The qRT-PCR program was set according to the manufacturer’s instructions (Wang et al., 2017). The 2^–ΔΔ*CT*^ method was used to determine the relative expression levels. The housekeeping gene, β*-actin* (No. HM640276), was used as an internal control to quantify relative transcript levels, which is stably expressed and has been chosen as the reference for many species, including *A. cerana cerana* (Lourenço et al., 2008). Each qRT-PCR experiment was repeated three times, with triplicates for each individual sample (*n* = 9). The sequences of the primers used for qRT-PCR are indicated in Supplementary Table 1.

### Bioinformatics Analysis

The UGT mRNAs of *A. cerana cerana* were obtained from the NCBI database^1^ using BLAST searches according to the UGT gene motifs (Supplementary Table 2). Multiple alignments of the AccUGT proteins were generated using DNAMAN version 7.^2^ The conserved motifs were analyzed using MEME.^3^ The UGT amino acid sequences of *Plutella xylostella* (*P. xylostella*) (Li X. et al., 2018), *Drosophila melanogaster* (*D. melanogaster*) (Ahn et al., 2012), *Helicoverpa armigera* (*H. armigera*) (Ahn et al., 2012), and *Bombyx mori* (*B. mori*) (Huang et al., 2008) were recognized from previous studies and downloaded from the NCBI database. The amino acid sequences of UGT genes from different species are given in Supplementary Table 3. Amino acid sequences of UGTs from *A. cerana cerana* and five other insects were analyzed via ClustalW alignment using MEGA7 software.^4^ The alignment results were used to build a consensus phylogenetic tree using the neighbor-joining method. A total of 1,000 bootstrap replications were performed, and branches with bootstrap values above 50% are indicated.

### RNAi of AccUGT2B20-Like

To silence the *AccUGT2B20-like* gene, double-stranded RNAs (dsRNA) were synthesized using the T7 RiboMAXTM Express RNAi System (Promega, United States) following the manufacturer’s instructions (Wang et al., 2018b). The dsRNA of the green fluorescent protein (GFP; GenBank accession No. U87974) sequence, which does not exist in the genome of bees, was used as a control. The experimental group was injected with 1 μl (6 μg/individual) of ds*AccUGT2B20-like*, and the control groups were untreated but injected with an equal amount of dsRNA-GFP and water (1 μl). dsRNA or water was injected into the third and fourth abdominal segments as described by Li G. et al. (2018). Silenced efficiency was determined by qRT-PCR. The silenced bees were used to analyze oxidant- and antioxidant-related genes and enzymes.

### Transcriptional Levels of Antioxidant-Related Genes After Silencing the AccUGT2B20-Like Gene

To explore the changes in antioxidant genes after *AccUGT2B20-like* was silenced, the expression levels of 10 antioxidant genes were analyzed by qRT-PCR. These 10 genes included *AccCAT* (GenBank ID: KF765424), *A. cerana cerana* cytochrome P450 (Acccyp4g11, GenBank ID: KC243984), *AccGrx1* (GenBank ID: JX844656), *AccGSTO1* (GenBank ID: KF496073), *AccGSTO2* (GenBank ID: JX434029), *AccGSTD* (GenBank ID: JF798572), *AccGSTS4* (GenBank ID: JN008721), *AccHsp22.6* (GenBank ID: KF150016), *AccSOD1* (GenBank ID: JN700517), and *AccTrx1* (GenBank ID: JX844651). These genes were selected based on our previous study (Zhang et al., 2016). The primers used in this experiment are shown in Supplementary Table 1.

### Determination of Antioxidant Enzyme Activity

Total protein was extracted from the silenced bees in a 1-ml isolation medium using the Tissue Protein Extraction Kit according to the manufacturer’s protocol. Total protein was quantified using the total protein assay kit, the BCA Protein Assay Kit (Nanjing Jiancheng Bioengineering Institute). Enzyme activities of superoxide dismutase (SOD), catalase (CAT), peroxidase (POD), and malondialdehyde (MDA) were detected using the SOD assay kit, CAT assay kit, POD assay kit, and MDA assay kit, respectively (all four kits were produced by Nanjing Jiancheng Bioengineering Institute, Nanjing, China).

### Protein Expression and Disc Diffusion Assays

To construct an *AccUGT2B20-like* recombinant, the *AccUGT2B20-like* open reading frame was amplified using the primers eUF and eUR, and inserted into an expression vector, pET-30a(+). Then, the expression plasmid pET-30(+)-*AccUGT2B20-like* was transformed into *E. coli* strain BL21 (DE3) for protein expression. The recombinant was induced with 0.4 mM isopropyl β-D-1-thiogalactopyranoside (IPTG) and grown at 37°C for 8 h. The recombinant protein was analyzed by 12% sodium dodecyl sulfate-polyacrylamide gel electrophoresis (SDS-PAGE), followed by Coomassie brilliant blue staining. To evaluate the activity of recombinant AccUGT2B20-like cells, disc diffusion assays were performed as described by Jia et al. (2017). *E. coli* cells overexpressing AccUGT2B20-like were overlaid onto LB-kanamycin agar and incubated at 37°C for 1 h. *E. coli* BL21 cells containing an empty pET-30a (+) vector were used as controls. Filter discs (6 mm diameter) soaked in different concentrations of cumene hydroperoxide (CHP) (0, 0.10, 0.14, 0.20, and 0.41 μmol) and tert-Butyl hydroperoxide (TBHP) (0, 0.16, 0.21, 0.32, and 0.64 μmol) were placed on the surface of agar. Following incubation at 37°C for 12 h, the killing zones around the paper discs were measured as described by Burmeister et al. (2008).

### Statistical Analysis

The results are presented as mean ± standard error of the mean (SEM) from three independent biological replicates. Statistical analysis was performed using SPSS (v. 25). Means were compared using Student’s t-test or one-way analysis of variance (ANOVA). Significance was set at ^∗^*P* < 0.05 and ^∗∗^*P* ≤ 0.01.

## Results

### Identification and Phylogenetic Analysis of AccUGT Genes

**FIGURE 1:**
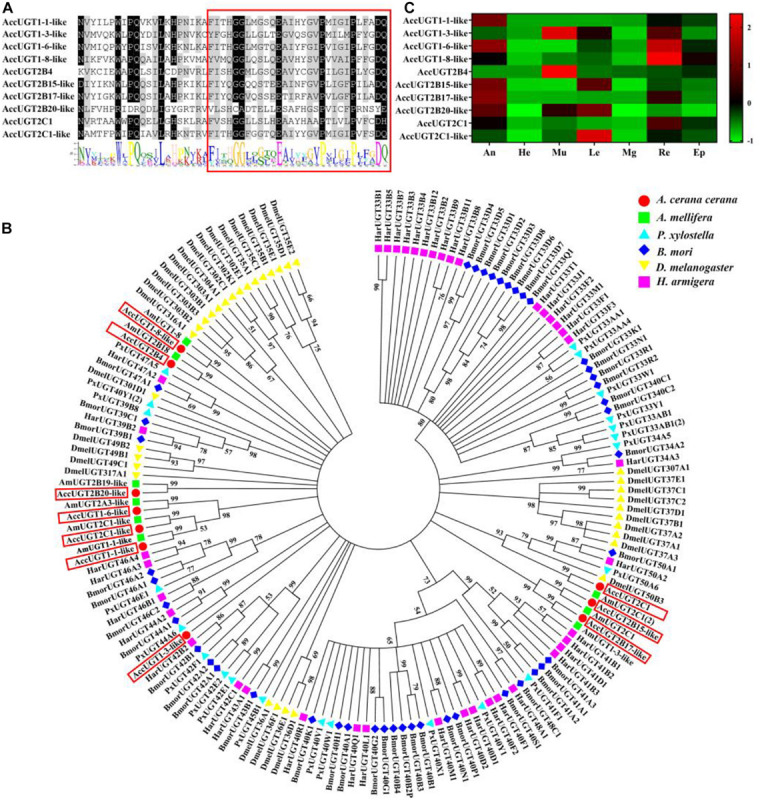
Phylogenetic analysis and tissue-specific expression patterns of *Apis cerana cerana* UDP-glucuronosyltransferases (UGT) genes. **(A)** Signature motif of *AccUGT* genes. Multiple alignment analysis of *A. cerana cerana*. The motif sequence discovered by MEME (http://meme-suite.org/) is shown at the bottom. The UGT signature motif is in the box. **(B)** The phylogenetic analysis involved 159 UGT amino acid sequences from *Apis cerana cerana* (10), *Apis mellifera* (9), *Plutella xylostella* (22), *Bombyx mori* (44), *Drosophila melanogaster* (34), and *Helicoverpa armigera* (40). Evolutionary analyses were conducted in MEGA7. **(C)** Tissue-specific expression patterns of *AccUGTs* from adult bees based on qRT-PCR. The mean expression value of each UGT gene was normalized using the Z-score method. He, head; An, antenna; Mu, muscle; Le, leg; Mg, midgut; Re, rectum; Ep, epidermis.

A signature sequence involved in the binding of the UDP moiety of the nucleotide sugar has been identified as a characteristic sequence in different organisms. To gain insight into the UGT family in *A. cerana cerana*, the amino acid sequence corresponding to this signature motif in insect UGTs was used to screen the predicted *A. cerana cerana* protein database. We ultimately identified 10 UGT genes from the *A. cerana cerana* genome, and the GenBank accession numbers are shown in Supplementary Table 2. Multiple alignments of 10 AccUGT amino acid sequences revealed the same signature sequence at the C-terminal (Figure 1A). The UGT motif signature sequence, (FVA)-(LIVMF)-(TS)-(HQ)-(SGAC)-G-X(2)-(STG)-X(2)-(DE)-X(6)-P-(LIVMFA)-(LIVMFA)-X(2)-P-(LMVFIQ)-X(2)-(DE)-Q, was also detected among the AccUGT genes. A phylogenetic tree constructed with amino acid sequences from *A. cerana cerana* (10), *Apis mellifera* (9), *P. xylostella* (22), *B. mori* (44), *D. melanogaster* (34), and *H. armigera* (40) revealed patterns of interspecific conservation and lineage-specific expansion of the gene families (Figure 1B). The 10 AccUGT genes were distributed into seven subfamilies, of which *AccUGT1-6-like*, *AccUGT2C-1-like*, and *AccUGT1-1-like* were clustered into one subfamily and *AccUGT2B15-like* and *AccUGT2B17-like* were clustered into another. The remaining five AccUGT genes were divided into five different subfamilies.

### Tissue-Specific Expression Patterns of AccUGT mRNAs

Tissue-specific expression patterns of 10 AccUGT genes were analyzed in adult bees by qRT-PCR. Among the different tissues, the antenna, muscle, and rectum had the highest level of expression. Five AccUGT genes exhibited high expression in the antenna and three did so in the muscle. Conversely, most UGTs exhibited lower expression in the head. Among the 10 UGT genes, *AccUGT2B4*, *AccUGT1-3-like*, *AccUGT2C1-1-like*, and *AccUGT2B20-like* exhibited high expression in different tissues, indicating they may have important regulatory functions. *AccUGT2B4* showed the highest expression in all tested tissues, especially in the muscle and epidermis (Figure 1C).

### Expression Profile of AccUGT Genes Under Different Pesticides and Oxidative Stress

**FIGURE 2:**
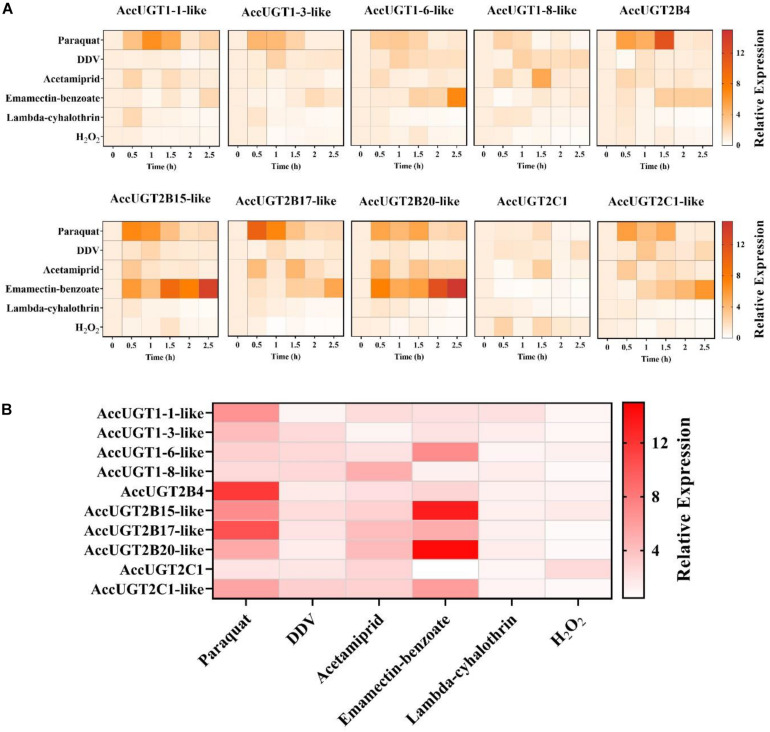
Expression pattern of *AccUGT* genes under different treatment conditions. **(A)** Expression profile of 10 AccUGT genes under pesticides and H_2_O_2_ stresses. **(B)** Most highly upregulated gene expression-fold change under different stresses. The most highly upregulated gene expression-fold change relative to that of the control (CK) group under different treatments. AccUGT mRNA expression was normalized using β*-actin* expression.

To explore whether UGT genes in *A. cerana cerana* respond to pesticides, five different pesticides and H_2_O_2_ treatments were used to evaluate the expression of UGT genes in adult worker bees. After pesticide treatment, we chose the most highly upregulated gene based on expression-fold change relative to that of the control group. Thus, we could screen for genes in the UGT gene family that had a stronger response to pesticide treatment. The qRT-PCR results indicated that almost all the transcripts of the 10 UGT genes were induced by our treatments, of which *AccUGT2B15-like*, *AccUGT2B17-like*, and *AccUGT2B20-like* were significantly upregulated. Specifically, *AccUGT2B20-like* was upregulated in emamectin-benzoate, acetamiprid, and lambda-cyhalothrin treatments (Figure 2A). Therefore, based on the results of tissue-specific analysis and expression profiling analysis, the *AccUGT2B20-like* gene was identified as the major gene involved in resistance against the pesticides. We further chose *AccUGT2B20-like* to conduct the following experiment to investigate the function of AccUGTs.

### Characterization of AccUGT2B20-Like

**FIGURE 3:**
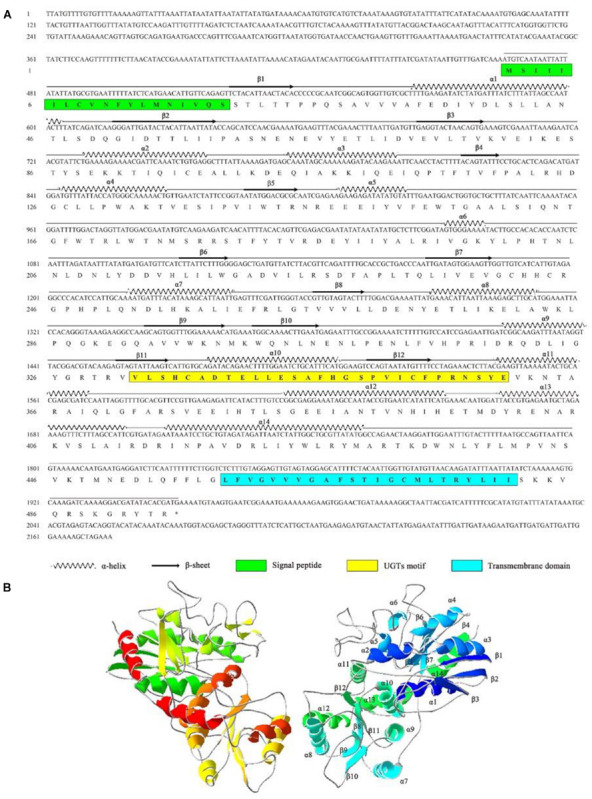
Characteristics of the *AccUGT2B20-like* gene. **(A)** cDNA and amino acid sequence of *AccUGT2B20-like*. The predicted protein secondary structure of AccUGT2B20-like is marked on the amino acid sequence. The predicted signal peptide in the N-terminus is in the green box, the UGT signature motif is in the yellow box, and the transmembrane domain in the C-terminal half and cytoplasmic tails are in the blue box. The sequence was deposited in GenBank (XM_017063111.1). **(B)** Putative tertiary structure characteristics of AccUGT2B20-like. The sequence was predicted using the SWISS-MODEL. The α-helices, β-sheets, coils were color-coded by secondary structure succession using SWISS-PDB VIEWER software, version 4.1.0 (Swiss Institute of Bioinformatics, Geneve, Suisse).

The full-length cDNA of *AccUGT2B20-like* (GenBank accession number XM_017063111) was 2,174 bp, which encodes a 494-amino acid protein with a predicted average mass of 56.804 kD. The full-length nucleotide and deduced amino acid sequences are shown in Figure 3A. The signal peptide was found at the N-terminal end of *AccUGT2B20-like*. The UGTs signature motif was found in the middle of the C-terminal domain, which exhibited higher conservation. A single short transmembrane domain, composed of 23 hydrophobic amino acid residues, occurred close to the end of the C-terminal domain. To further elucidate the protein structure of AccUGT2B20-like, we predicted its tertiary structure using the online database SWISS-MODEL (Figure 3B). We detected 14 α-helices and 12 β-sheets in AccUGT2B20-like, and the tertiary structure of proteins will provide strong evidence for our understanding of protein function.

### Expression Profile of AccUGT2B20-Like Under a Variety of Pesticides

**FIGURE 4:**
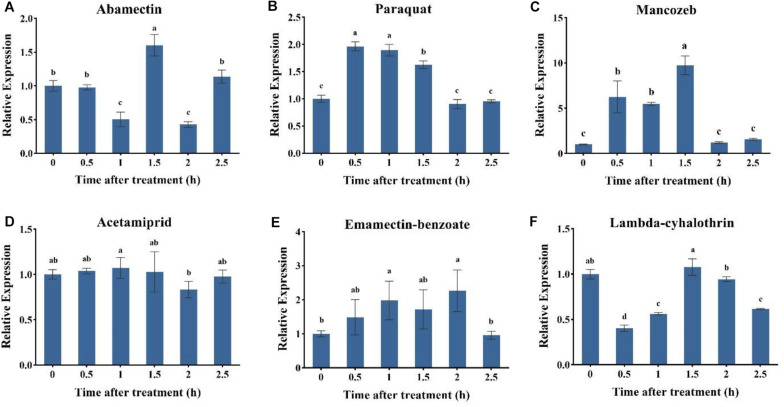
Expression profiles of the *AccUGT2B20-like* gene under different pesticides. qRT-PCR was performed on the total RNA extracted from adult bees at the indicated time points following the different stresses. The stressors include **(A)** abamectin, **(B)** paraquat, **(C)** acetamiprid, **(D)** acetamiprid treatment, **(E)** emamectin-benzoate, and **(F)** lambda-cyhalothrin treatment. The vertical bars represent the mean ± SEM (*n* = 9). β*-actin* (GenBank accession number: HM640276) was used as the control. The letters above the bars indicate significant differences (*P* < 0.05 in SPSS) based on Duncan’s multiple range test.

To investigate the specific role of *AccUGT2B20-like* under different pesticides, the expression profile of *AccUGT2B20-like* was examined by qRT-PCR. As shown in Figure 4, among six pesticide treatments, the application of paraquat, mancozeb, emamectin-benzoate, and lambda-cyhalothrin significantly induced the expression of *AccUGT2B20-like*. The response time was short, and the expression level of *AccUGT2B20-like* reached its peak within 2 h. *AccUGT2B20-like* was significantly upregulated under paraquat, acetamiprid, and lambda-cyhalothrin treatment, which was significantly different from that of the control group. The above results showed that *AccUGT2B20-like* is involved in protecting bees against damage from different pesticides.

### Disk Diffusion Assays of Recombinant AccUGT2B20-Like in Response to Oxidative Stress

**FIGURE 5:**
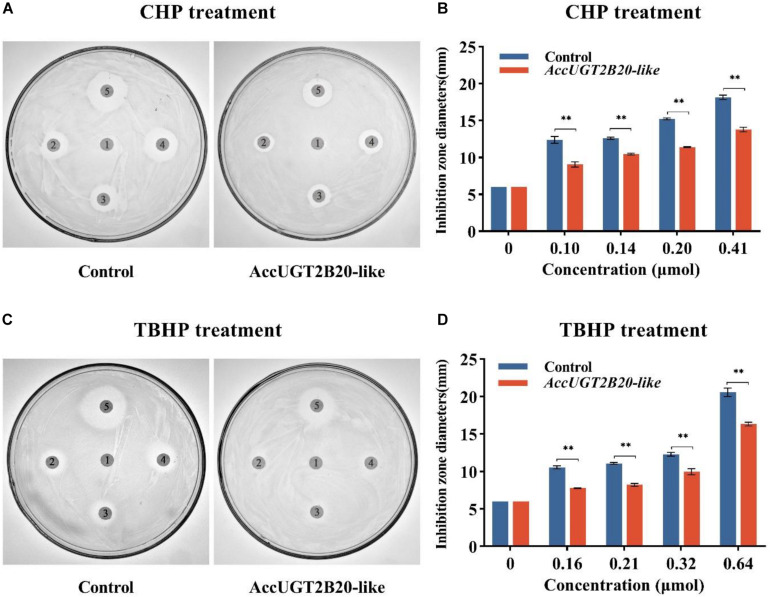
Disc diffusion assays using *E. coli* overexpressing *AccUGT2B20-like*. LB agar plates were inoculated with 5 × 108 cells. *AccUGT2B20-like* cells were overexpressed in *E. coli*, and bacteria transfected with pET-30a (+) were used as negative controls. Filter discs soaked with different concentrations of CHP **(A,B)** and TBHP **(C,D)** were placed on the agar plates. After overnight exposure, the killing zones around the drug-soaked filters were measured. *P < 0.05; **P < 0.01.

To explore the molecular regulation mechanism of AccUGTs in response to oxidative stress, we overexpressed AccUGT2B20-like in *E. coli*, and disk diffusion assays were conducted. After overnight exposure to cumene hydroperoxide (CHP) and tert-butyl hydroperoxide (TBHP), the killing zones around the drug-soaked filters were smaller on the plates containing *E. coli* overexpressing AccUGT2B20-like than on that of the control bacteria that were transfected with the vector only (Figure 5). These results provided further evidence that overexpression of AccUGT2B20-like can effectively improve resistance to oxidative stress.

### Effects of AccUGT2B20-Like Knockdown on Oxidative Stress

**FIGURE 6:**
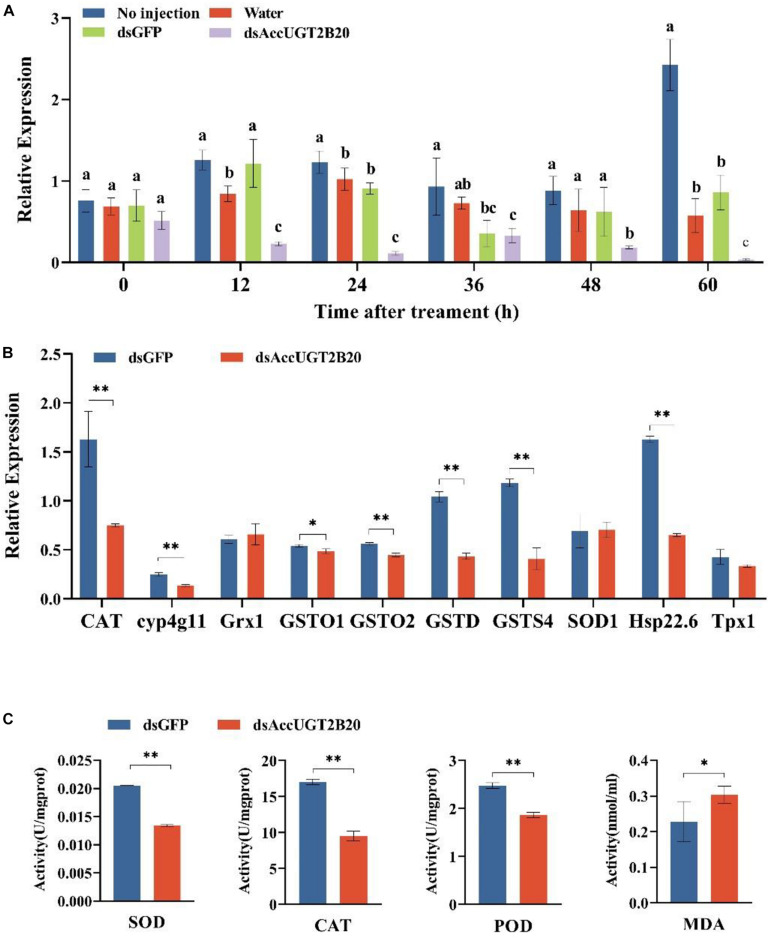
Effects of the RNAi-mediated silencing of *AccUGT2B20-like*. **(A)** qRT-PCR confirmation of RNAi knockdown of *AccUGT2B20-like*. **(B)** Expression patterns of antioxidant genes at 24 h after *AccUGT2B20-like* knocked down, as analyzed by qRT-PCR. **(C)** Determination of antioxidant enzyme activity. The error bars indicate 95% confidence intervals. ^∗^*P* < 0.05; ^∗∗^*P* < 0.01.

To further investigate the regulatory function of AccUGTs in response to oxidative stress induced by pesticides, we employed RNAi to knock down the expression of *AccUGT2B20-like*. dsRNA specific to *AccUGT2B20-like* was microinjected into adult workers to silence the target gene. For controls, adult workers were microinjected with dsGFP or water. qRT-PCR was used to detect the expression level of *AccUGT2B20-like* and determine the optimal silencing time. The transcription level of *AccUGT2B20-like* in the dsAccUGT2B20-injection group was significantly inhibited compared with that of the control groups, especially at 24 h (Figure 6A). The results indicated that dsRNA-mediated gene silencing was successful, and 24 h after dsRNA injection was selected for further study.

To elucidate the effects of *AccUGT2B20-like* knockdown, the transcriptional levels of other antioxidant genes were also investigated by qRT-PCR. We analyzed the expression levels of 10 different antioxidant genes 24 h after *AccUGT2B20-like* was silenced (Figure 6B). The results showed that *AccCAT*, *Acccyp4g11*, *AccGSTO1*, *AccGSTO2*, *AccGSTD*, *AccGSTS4*, and *AccHsp22.6* were downregulated when *AccUGT2B20-like* was silenced. To confirm the effects of RNAi with *AccUGT2B20-like* on the antioxidant capacity of *A. cerana cerana*, we measured the enzymatic activities of SOD, CAT, POD, and MDA after RNAi treatment for 24 h. The activities of SOD, CAT, and POD declined compared with that of the control groups. Furthermore, MDA concentration increased after the *AccUGT2B20-like* expression was knocked down (Figure 6C). These results indicated that the knockdown of *AccUGT2B20-like* increased oxidative stress in *A. cerana cerana*.

## Discussion

In recent years, because of various environmental stresses, including pesticides, the number of *A. cerana cerana* has gradually decreased. However, the specific molecular mechanism of the resistance of Chinese honeybees to pesticide stress is still unclear. UGTs, as significant major phase II drug-metabolizing enzymes, play important roles in the detoxification process (Bock, 2003). Many UGT genes have been identified and exist in almost all living organisms. The UGT gene family is particularly interesting because it is involved in human disease, drug metabolism, and homeostasis maintenance (Rowland et al., 2013; Bock, 2016; Mazerska et al., 2016). However, information regarding the functions of AccUGT genes remains limited.

In this study, we identified the UGT genes in *A. cerana cerana* and investigated the molecular regulatory mechanism of UGTs in oxidative stress induced by pesticides. UGT genes have been identified in insects, such as *D. melanogaster* (Morello and Repetto, 1979), *B. mori* (Huang et al., 2008), *Spodoptera littoralis* (Bozzolan et al., 2014), *H. armigera* and *Heliothis virescens* (Krempl et al., 2016), and *Aphis gossypii* (Pan et al., 2018). Many studies have been conducted on the UGTs in insects; nevertheless, a few studies have been conducted using *A. cerana cerana*. In the present study, we identified 10 UGT sequences from the *A. cerana cerana* cDNA database using the UGT motif signature (Figure 1A). The C-terminal is highly conserved where the UGT motif signature is found. This result is consistent with that of Meech and Mackenzie (1998). Compared to that of other insects, *A. cerana cerana* has a lower number of UGT genes. This may be related to the highly specialized habitat, lack of exposure to food-derived toxins, and peculiar life history (Honeybee Genome Sequencing Consortium, 2006).

Understanding the tissue-specific expression patterns of AccUGTs may provide a better understanding of functional mechanisms. Therefore, we analyzed the relative expression of UGT genes in different tissues (Figure 1C). As mentioned in the literature, insect UGTs are preferentially expressed in the olfactory organ, the antenna, suggesting that UGTs may be involved in the deactivation of pheromones (Wang et al., 1999; Younus et al., 2014). Our results agree with those observed in earlier studies. Among 21 UGTs in *P. xylostella*, 8 exhibited high expressions in the midgut and 3 in the fat body (Li X. et al., 2018). The insect midgut is the largest part of the digestive tract and is the main target of pathogenic microorganisms, pesticides, and plant toxins (Li et al., 2008). In this study, the midgut did not exhibit high expression; however, higher gene expression was detected in the rectum. This result may be related to the method of injection. We injected the pesticide directly into the abdomen of the bees, but in their natural state, bees feed on pesticide-containing pollen or nectar, so the high expression of UGT in the midgut is reasonable. UGT genes with high relative expression in various tissues may play a critical role in honeybees. Highly expressed UGT genes, such as *AccUGT2B4* and *AccUGT2B20-like*, may be candidates for the screening of major genes.

Previous studies have shown that the UGT genes are involved in the detoxification process of insects and respond to different pesticides or insecticides (Ahn et al., 2012; Bock, 2016). In a multiresistant population of *P. xylostella*, 10 of 21 UGT genes were upregulated, and the population was resistant to nine commonly used insecticides. The expression of *UGT40V1*, *UGT45B1*, and *UGT33AA4* could be induced by more than five insecticides (Li X. et al., 2018). Comparison analysis of thiamethoxam-resistant cotton aphids and susceptible aphids showed that 13 UGT genes were increased by approximately 2.0-fold or greater in the thiamethoxam-resistant strain (Pan et al., 2018). According to previous research, we proposed that the UGT family may contribute to the protection of *A. cerana cerana* from pesticides. In this study, almost all the UGT genes were upregulated after exposure to different pesticides (Figure 2A). These results indicated that UGT genes are involved in the process of pesticide response in *A. cerana cerana*.

The UGT2B enzymes are important in the detoxification of a variety of endogenous and exogenous compounds. Previous studies have demonstrated that UGT2B15 exhibits high activity of BPA glucuronidation and is closely associated with the metabolism and toxicity of BPA, an endocrine-disrupting chemical that could result in reproductive and developmental toxicity at low doses (Richter et al., 2007; Hanioka et al., 2011). A study by Li et al. (2017) proved that overexpression of *UGT2B17* decreased the toxicity of chlorantraniliprole and may play a critical role in chlorantraniliprole resistance in *P. xylostella*. However, the detoxification of UGT2B20 has not yet been reported. *A. cerana cerana* was subjected to 8 toxicity stressors in this study; all 10 UGT mRNAs were induced, among which the AccUGT2B family gene was significantly upregulated (Figure 2B). This result indicates that the UGT2B family may play an essential role in the resistance of the *A. cerana cerana* to external stresses. Interestingly, *AccUGT2B20-like* was induced in five of the eight treatments, and it was the most significantly upregulated gene under different treatments. We consequently chose *AccUGT2B20-like* as a representative of this gene family to explore their properties.

Many studies have demonstrated that pesticides, including insecticides and herbicides, can induce oxidative stress, leading to alteration in lipid peroxidation, free-radical scavengers, and a series of antioxidants (Banerjee et al., 1999; Etemadi-Aleagha et al., 2002; Abdollahi et al., 2004). Additionally, some studies have shown that UGTs are involved in the detoxification processes, including that of drugs, pesticides, and endogenous compounds (Mazerska et al., 2016). Therefore, to explore the relationship between UGTs and oxidative stress, we simulated several adverse life-threatening environmental conditions that could cause ROS damage in *A. cerana cerana*. Furthermore, we explored the molecular regulation mechanism of UGTs in response to oxidative stress. Previous studies have shown that overexpression of antioxidant-related genes, such as *AccSCO2*, *AccGSTO2*, and *AccTpx4* in *E. coli* cells could protect them from oxidative stressors by performing disc diffusion assays (Huaxia et al., 2015; Zhang et al., 2016; Jia et al., 2017). In this study, we overexpressed *AccUGT2B20-like* in *E. coli* cells, and the results were consistent with those of earlier studies. Overexpression of *AccUGT2B20-like* could protect *E. coli* from oxidative stressors (Figure 5). This result provides preliminary evidence for the antioxidant function of UGTs.

High ROS concentrations in cells can cause oxidative stress and contribute to degenerative diseases and aging through the oxidation of nucleic acids, proteins, and lipid membranes (Jia et al., 2014). As byproducts of aerobic metabolism, ROS continuously exists during oxidative stress, whereas some antioxidant genes, such as those for SOD, ascorbate peroxidase (APX), glutathione peroxidase (GPX), and CAT, could serve as detoxication for ROS elimination (Apel and Hirt, 2004). Using antioxidant-related genes as marker genes to detect their expression can indirectly reflect their antioxidant properties. Previous reports have suggested that when *AccGSTO2* is knocked down, most antioxidant-related gene expression is reduced (Zhang et al., 2016). Wang reported that *AccSOD1*, *AccSOD2*, *AccGSTS4*, *AccCYP4G11*, *AccGSTO2*, and *AccGSTD* was downregulated when *AccMKK6* was knocked down (Wang et al., 2018b). To further investigate the gene function of *AccUGT2B20-like* and the relationship between *AccUGT2B20-like* and oxidation-related genes, *AccUGT2B20-like* gene was silenced. Then, we find that the expression levels of antioxidant genes were significantly reduced. This result suggested that *AccUGT2B20-like* may participate in the regulation of downstream antioxidant genes. The function of a gene is achieved through its expression product. Therefore, determining the activities of antioxidant enzymes will reflect the real function. After UGT enzymes were inhibited by two inhibitors (sulfinpyrazone and 5-nitrouracil), resistance to thiamethoxam by aphids was decreased (Pan et al., 2018). The activities of SOD, POD, and CAT were reduced, whereas MDA was increased in silenced bees (Figure 6C). This result indicated that the oxidative damage to the body was increased. Considering our results in the context of previous studies, we concluded that AccUGTs are involved in the detoxification process of pesticides. Furthermore, *AccUGT2B20-like* possesses potent antioxidant properties that contribute to the detoxification of pesticides by eliminating oxidative stress.

## Conclusion

Our results indicate that UGTs are involved in pesticide resistance in *A. cerana cerana*, among which, *AccUGT2B20-like* contributes to the detoxification of pesticides by eliminating oxidative stress in *A. cerana cerana*. This study explains the molecular basis for the resistance of bees to pesticides and provides an essential safeguard for maintaining ecological balance.

## Data Availability Statement

All datasets generated for this study are included in the article/Supplementary Material, further inquiries can be directed to the corresponding authors.

## Ethics Statement

The animal study was reviewed and approved by the Committee on Animals of Shandong Agricultural University Animal Care and Use Committee (SDAUA-2018-018).

## Author Contributions

XC was involved in the experimental design, data processing, and manuscript writing. CW, BX, and XG were involved in the experimental design and constructive amendments to the manuscript. XW participated in the qRT-PCR and gene silencing work. GL, ZL, and HW helped in the editing of the manuscript. All authors read and approved the final manuscript.

## Conflict of Interest

The authors declare that the research was conducted in the absence of any commercial or financial relationships that could be construed as a potential conflict of interest.

## Footnotes

1 https://www.ncbi.nlm.nih.gov/

2 https://www.lynnon.com

3 http://meme-suite.org/tools/meme

4 https://www.megasoftware.net/
